# Supplementary material for: Breast cancers that disseminate to bone marrow acquire aggressive phenotypes through CX43-related tumor-stroma tunnels
Source: J Clin Invest. 2024 Oct 31;134(24):e170953. doi: 10.1172/JCI170953 (PMC11645149; doi:10.1172/JCI170953)

Full unedited blot for Figure 4B

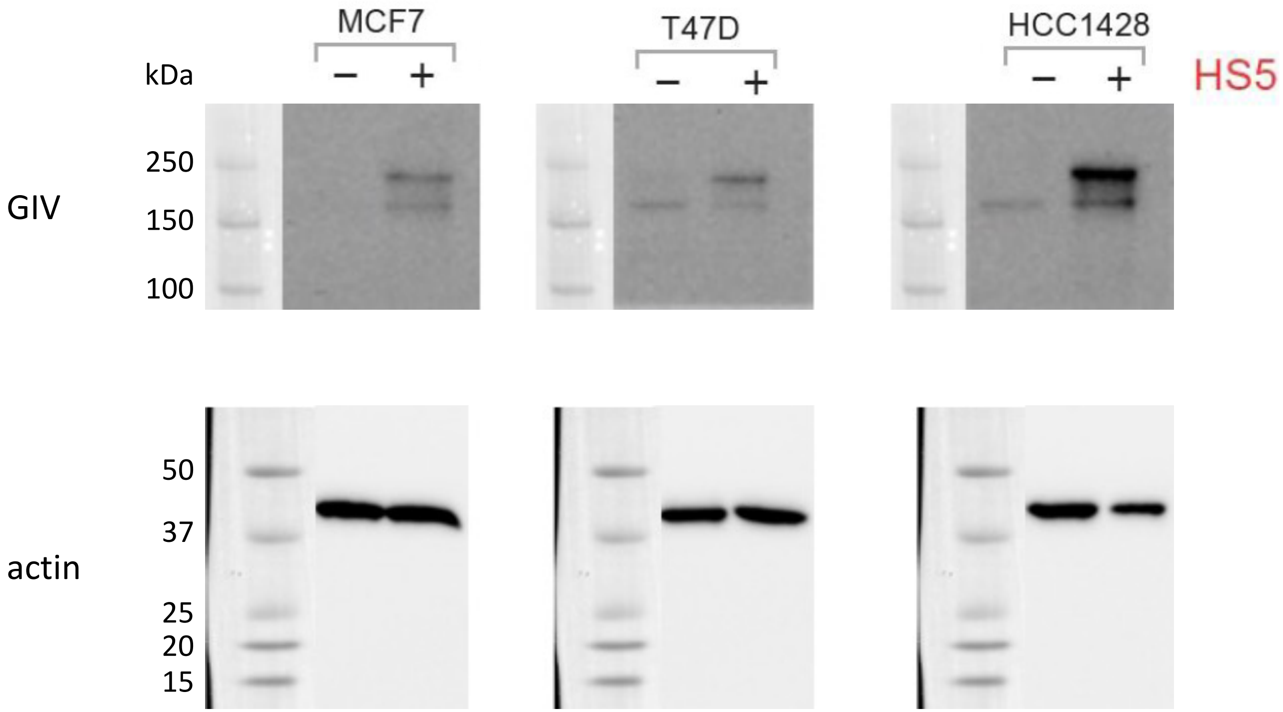

Molecular weight marker, colorimetric image; labeled lanes, chemiluminescent image. All lanes on the blot correspond with the cropped lanes and images shown in Figure 4B.

Full unedited blot for Figure 4C

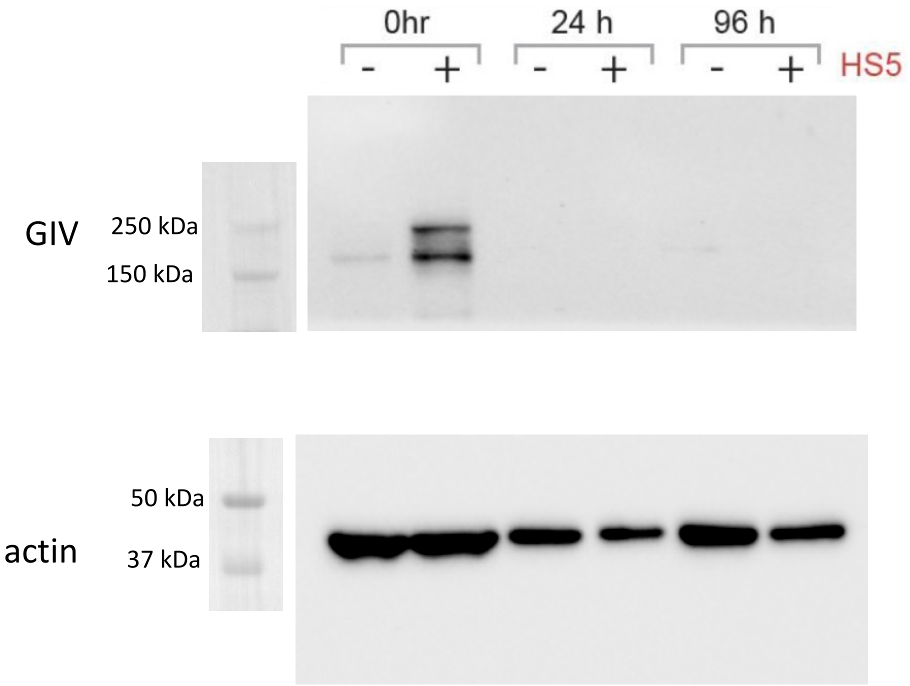

Molecular weight marker, colorimetric image; labeled lanes, chemiluminescent image.
All lanes on the blot correspond with the cropped lanes and images shown in Figure 4C.

Full unedited blot for Figure 4E

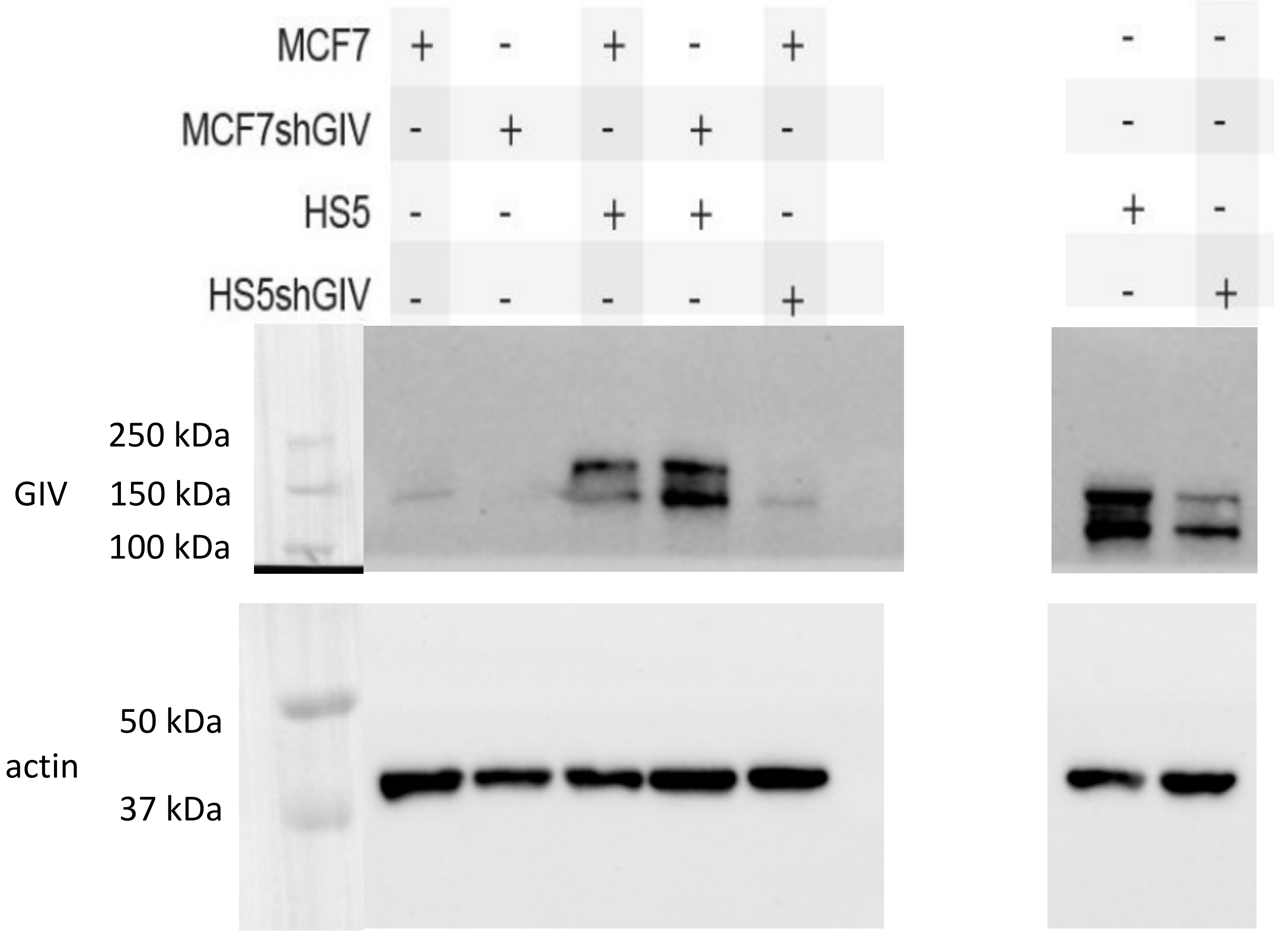

Molecular weight marker, colorimetric image; labeled lanes, chemiluminescent image.
All lanes on the blot correspond with the cropped lanes and images shown in Figure 4E.

Full unedited blot for Figure 5A

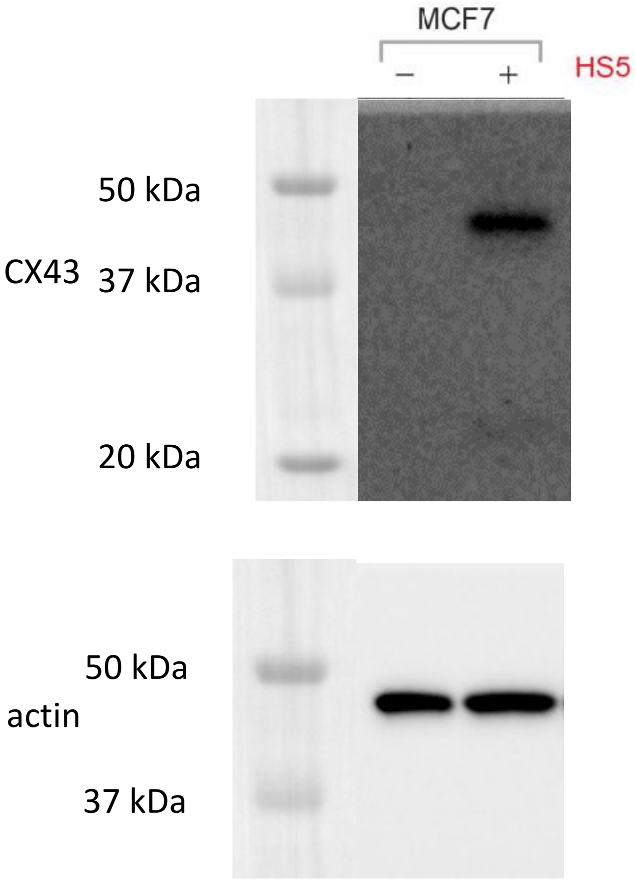

Molecular weight marker, colorimetric image; labeled lanes, chemiluminescent image.
All lanes on the blot correspond with the cropped lanes and images shown in Figure 5A.

Full unedited blot for Figure 5B

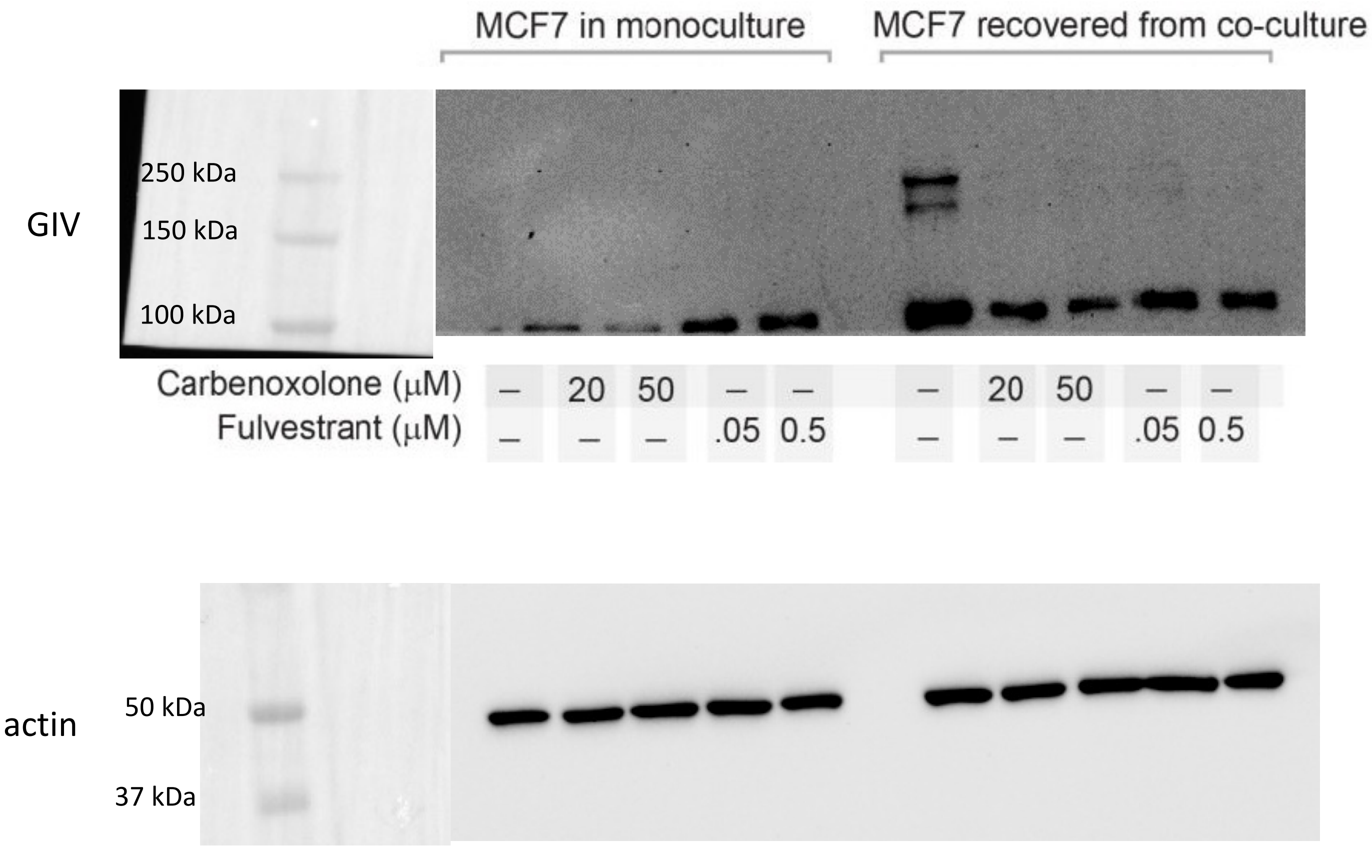

Molecular weight marker, colorimetric image; labeled lanes, chemiluminescent image.
All lanes on the blot correspond with the cropped lanes and images shown in Figure 5B.

Full unedited blot for Figure 5E

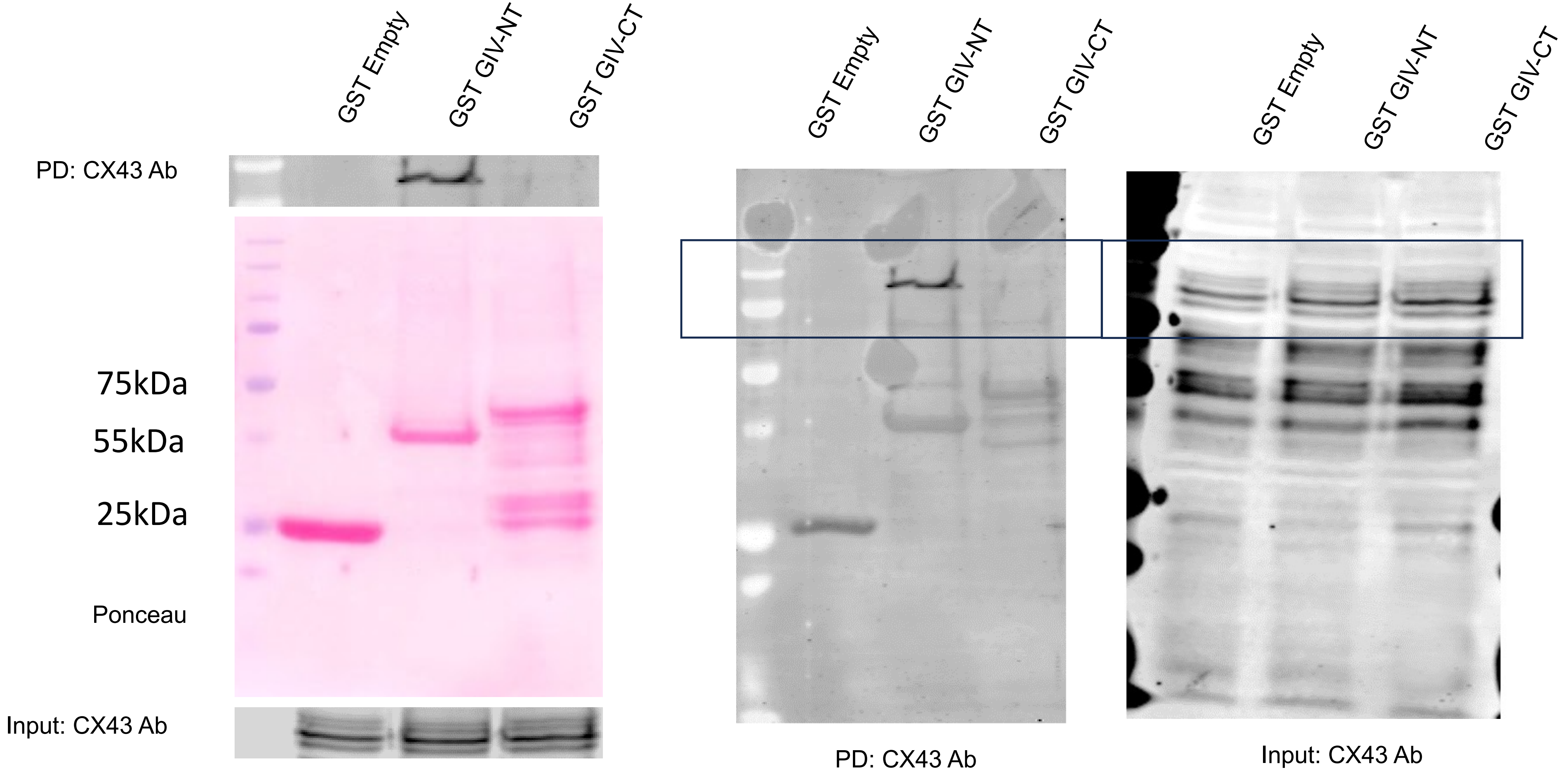

Pull-down assays using recombinant GST-tagged GIV N- or C-term fragments or GST alone

48 hrs transfection with CX43_GFP Plasmid. (Transfection done in Cos 7 cells.)

Full unedited blot for Figure 5E

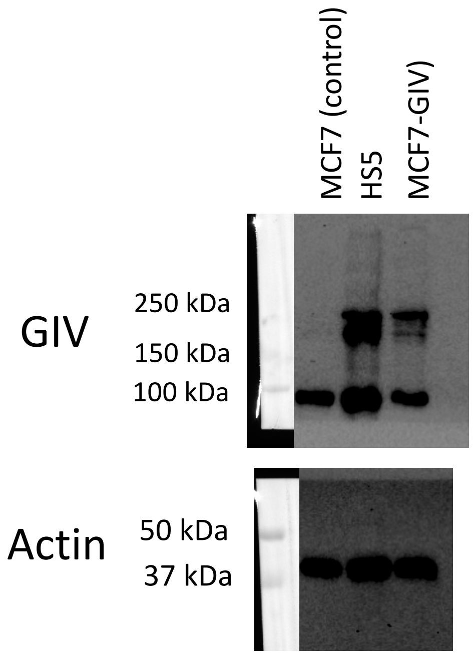

Molecular weight marker, colorimetric image; labeled lanes, chemiluminescent image.
All lanes on the blot correspond with the cropped lanes and images shown in Figure 5E.

Full unedited blot for Supplemental Figure 5

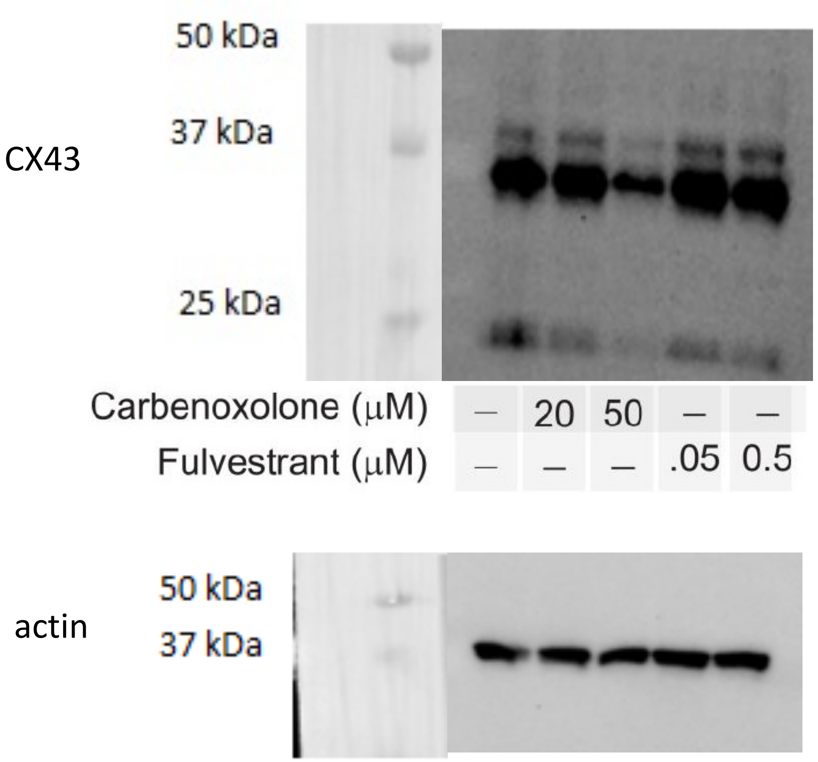

Molecular weight marker, colorimetric image; labeled lanes, chemiluminescent image.
All lanes on the blot correspond with the cropped lanes and images shown in Supplemental Figure 5.

Full unedited blot for Supplemental Figure 6

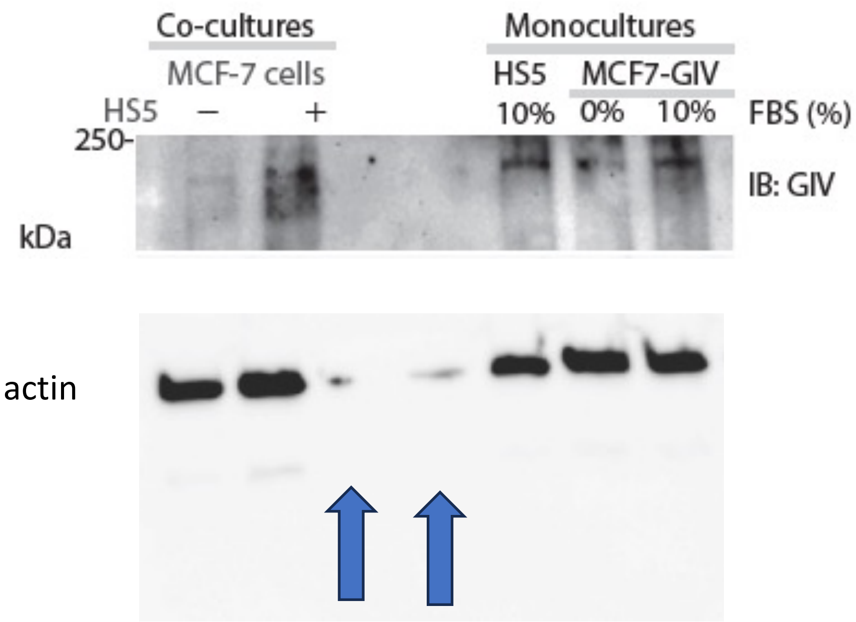

Empty lanes with small overflow from adjacent lanes

Blot presented in Supplemental Figure 6 for GIV is the full unedited version (reproduced here for comparison with the edited actin blot shown below).

Full unedited blot for Supplemental Figure 7

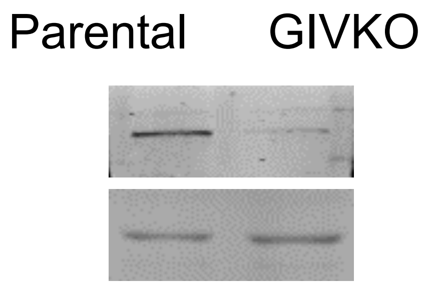

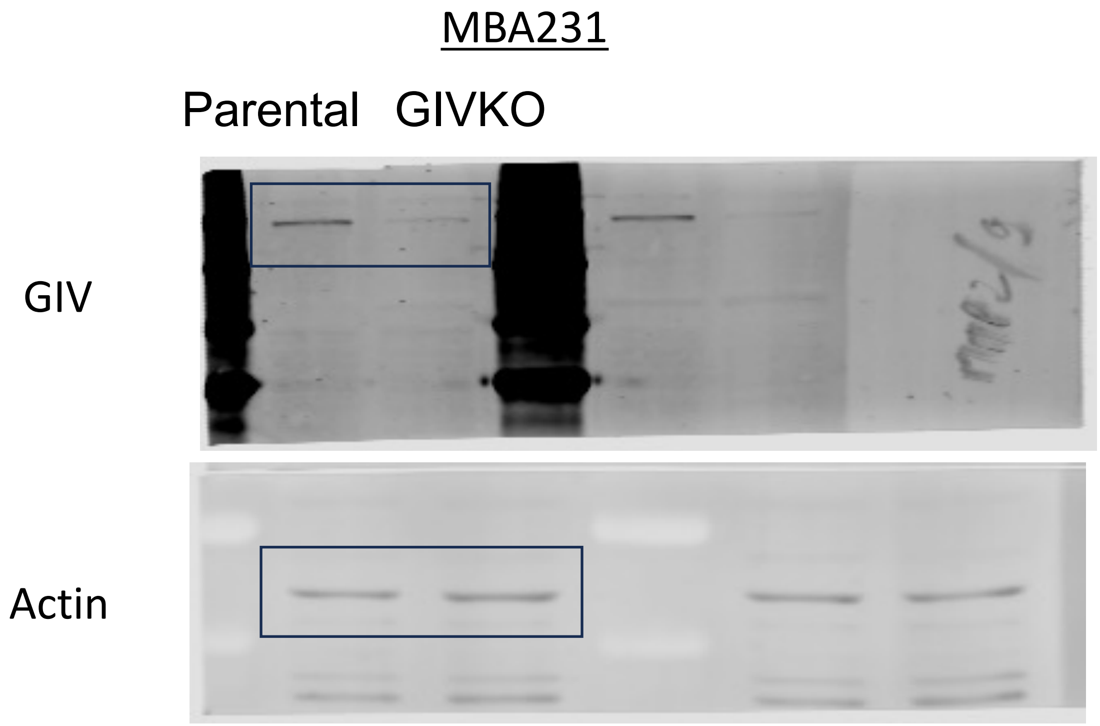

Supplement: Unedited blot and gel images [file jci-134-170953-s125.pdf]
